# Well-temperate phage: optimal bet-hedging against local environmental collapses

**DOI:** 10.1038/srep10523

**Published:** 2015-06-02

**Authors:** Sergei Maslov, Kim Sneppen

**Affiliations:** 1 Biological, Environmental and Climate Sciences Department, Brookhaven National Laboratory, Upton, NY 11973, USA; 2 Center for Models of Life, Niels Bohr Institute, University of Copenhagen, 2100 Copenhagen, Denmark

## Abstract

Upon infection of their bacterial hosts temperate phages must chose between lysogenic and lytic developmental strategies. Here we apply the game-theoretic bet-hedging strategy introduced by Kelly to derive the optimal lysogenic fraction of the total population of phages as a function of frequency and intensity of environmental downturns affecting the lytic subpopulation. “Well-temperate” phage from our title is characterized by the best long-term population growth rate. We show that it is realized when the lysogenization frequency is approximately equal to the probability of lytic population collapse. We further predict the existence of sharp boundaries in system’s environmental, ecological, and biophysical parameters separating the regions where this temperate strategy is optimal from those dominated by purely virulent or dormant (purely lysogenic) strategies. We show that the virulent strategy works best for phages with large diversity of hosts, and access to multiple independent environments reachable by diffusion. Conversely, progressively more temperate or even dormant strategies are favored in the environments, that are subject to frequent and severe temporal downturns.

Bacteria and their main predators, bacteriophages^1^,^2^, are the most abundant and dynamic part of the biosphere^3^,^4^. Phages lead a risky lifestyle^1^,^5^,^6^ and as a consequence local populations of individual phage species routinely experience extreme fluctuations caused by changes in availability of susceptible hosts^7^,^8^. In real ecosystems these fluctuations may be caused e.g. by depletion of nutrients for hosts, development of host resistance, and interference from competing phages^9^ or other bacterial predators.

Phages deal with these challenges using a variety of strategies^1^,^5^. One common strategy adopted by virulent phages is to always kill and lyse their host resulting in release of 100-3000 progeny phages 10. Sustainability of this strategy is critically dependent on phages’ ability to reach susceptible hosts 1,6,7,10,11,12 because free phage particles have a finite lifetime 10. In contrast, temperate phages following the infection of a bacterium can opt for a transition to the lysogenic state where the future fate of the incorporated prophage is aligned with that of its bacterial hosts. Ref. 13 suggested that the temperate strategy may persist because it allows phages to survive extended periods when host density is below the level needed to sustain the propagation of the lytic population (pure virulent strategy).

Campbell^1^ considered the sustainability of pure virulent strategy, noting that it strongly depends on the growth rate of the phages’ bacterial host relative to that of other competing bacteria in the same local environment. Thus, any changes in the environment affecting the relative ranking of bacteria by their growth rate will likely impact the local success of lytic phages infecting these bacteria^9^.

The lysogenic state of temperate phages allows them to weather out severe downturns in environmental conditions^13^,^14^. However, it comes at the cost of some reduction in the growth rate of the lytic subpopulation. It is hence plausible that temperate phages should try to optimize the ratio of their populations in lytic and lysogenic states in order to achieve the maximal long-term growth rate. To quantify this process we consider a local phage population living in a fluctuating environment characterized by sudden unpredictable downturns, a scenario inspired by the classical paper by Kelly^15^ on application of information theory to gambling. Like gamblers^15^ or financial investors^16^ phages must decide what part of their population “capital” to allocate to a “risky” lytic state which has the potential for rapid growth but is also subject to a non-negligible risk of sudden collapse. The rest of the population “capital” will be allocated to the relatively “safe” lysogenic state. Here we aim to quantify the parameters of the long-term optimal or “well-temperate” phage strategy and compare its outcome to a purely virulent strategy.

Our calculations are inspired by the original work by Kelly^15^, its applications to finance^16^,^17^ and evolution^18^. Similar bet-hedging approaches have been explored to germination of seeds from annual plants^19^,^20^,^21^. As is common for bet-hedging in population biology, our analysis goes beyond optimization of the outcome of individual infection on a short-time scale, and instead considers the long-term (logarithmic) growth rate^19^,^22^.

## Results

### The Kelly-optimal frequency of lysogeny

Consider a local phage population that grows or declines in fluctuating environmental conditions. The environment is assumed to randomly switch between “good” conditions favoring multiplicative growth of the lytic subpopulation and “bad” conditions during which local lytic subpopulation completely (or partially as will be investigated later in this study) dies out. Rapid growth during good conditions is quantified by the amplification factor Ω>1. We assume bad conditions to be transient events of indefinite duration that occur with the probability 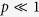. During good environmental conditions one time step of our model roughly corresponds to one phage generation which in turn makes Ω bounded from above by phage’s burst size. Conversely, during bad environmental conditions we count the entire duration of this event as a single time step.

Temperate phages in our model have no predictive knowledge of when their environment is about to turn bad. However, they are free to choose what fraction *x* of their population will be kept in the lysogenic state at every time step. Notice that because only during new infections by phages from the lytic subpopulation they are free to chose between lytic and lysogenic states, our model is based on the assumption that the majority of phages are of this type. This assumption is justified in case of rapid exponential growth when the lysogenization frequency during the last “good” time-step approximately determines the lysogenic fraction *x* for the entire population. In the simplest scenario considered in this chapter we also assume that the local lysogenic subpopulation is fully protected from the extreme changes in the local environment and does not change with time. Later on in this study we will relax this assumption and allow the lysogenic population to be characterized by a time-independent growth (or decline) rate. In fact the growth rate of the lytic subpopulation is always defined *relative* to that of the lysogenic subpopulation much in the same way as in financial markets risky asset returns are always compared to interest rates paid by banks. The expected value of the (logarithmic) growth rate Λ of the entire local phage population in our model is given by
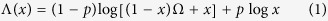
The first term is the logarithmic growth rate under good conditions when the lytic fraction 1 − *x* of the total population is multiplied by Ω, while the lysogenic subpopulation *x* remains unchanged. The second term is the logarithmic growth rate under bad conditions when only phages in the lysogenic state survive. Later on we will relax the requirement that the entire lytic population has to completely die off during bad times. The growth rate considered above weights the *logarithms* of multiplicative growth factors of the entire phage population under two conditions with their respective probabilities of occurrence. Maximization of Λ with respect to *x* secures the long-term optimal growth rate^15^. This should not be confused with optimization of the expected (average) population growth after just one or a small number of growth cycles. Such short-term optimization would always favor purely lytic strategy with *x* = 0 provided that (1 − *p*)Ω > 1. The last condition is almost always fulfilled since during good times Ω approaches its upper bound given by the average burst size which is substantially larger than one offspring per phage. On the other hand, following the lytic strategy for a long time would almost certainly bring the phage population to the total collapse, which will happen during the very first bad time interval.

**Figure 1:**
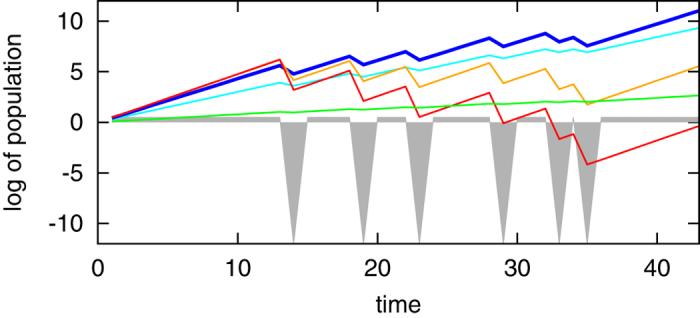
Phage population dynamics when exposed to long periods of exponential growth with Ω = 3 interrupted by occasional bad conditions where lytic subpopulation drops nearly to zero. Note the logarithmic scale base 10 on the y-axis. Bad conditions of severity *ω* = 10^−12^ happening with probability *p* = 0.1 are marked with downward-facing grey triangles. The blue curve is the growth of the phage population following the Kelly-optimal strategy with lysogenic fraction x* = *p* ⋅ Ω/(Ω − 1) = 0.15, whereas orange and red curves show suboptimal strategies with *x* = 0.01 and *x* = 0.001 correspondingly. Conversely, cyan and green curves simulate phage population dynamics with higher-than-optimal lysogenization frequencies of respectively *x* = 0.5 and *x* = 0.9. In the long run, phages following the Kelly-optimal strategy outperform their competitors. Note that the growth rate of the entire phage population is measured relative to that of the lysogenic subpopulation. If the latter is negative one can have a steady state Kelly-optimal solution (as opposed to the unlimited exponential growth shown here).

In contrast to its short-term counterpart, the long-term logarithmic growth rate Λ(*x*) usually reaches its maximum at some x* between 0 and 1. In the economics literature it is referred to as Kelly-optimal investment ratio^15^. It describes the optimal fraction of capital that a prudent long-term investor should keep in relatively safe financial assets such as bonds or bank deposits while investing the rest in more risky assets such as stocks^16^. In our biological interpretation x* corresponds to the optimal fraction of the phage population in the lysogenic state. At the Kelly-optimum the derivative of Λ with respect to *x* is equal to zero, which is realized at
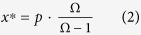
Figure 1. shows the dynamics of the total phage population at different values of x including its Kelly-optimal value (the blue curve). This equation is nearly identical to the Eq. 8 in^19^ which expresses the optimal germination frequency of plant seeds in environments where bad years would eliminate all germinated seeds. Furthermore, the positive correlation between x* and the frequency of environmental fluctuations *p* predicted by the Eq. (2) was confirmed by the empirical data on seed germination^19^,^23^.

Before we proceed with analysis and modifications of our model we would like to highlight the main approximations/simplifications used throughout this study. Our first approximation is the use of the Kelly theory, which implicitly assumes stochastic exponential growth. Thus in our simplified two-state model we ignore the (very real) dynamical feedback between populations of phages and their bacterial hosts as the former approach and ultimately reach steady state equilibrium. Such feedback in a system consisting of two types of phages (temperate and virulent) and one type of bacterial host (with susceptible, lysogenic, and resistant subpopulations) subject to variable nutrients was analyzed in a classic paper^13^. Stewart and Levin also compared purely virulent and temperate phage strategies in the steady-state of this closed system and reached general conclusions, which are in qualitative agreement with our predictions: “Lysogeny is an adaptation for phage to maintain their populations in “hard times”, when the host bacterial density oscillates below that necessary for phage to be maintained by lytic infection alone.” Here we confirm this prediction using a very different approach, and expand it by calculating the optimal lysogenic fraction of a temperate phage. Later on we will generalize our simplified two-state model characterized by the generic “good” or “bad” conditions to a multi-state model in which the current growth rate Ω(*t*) is drawn from an arbitrary (even continuous) probability distribution. This variant of the model in principle allows us to consider the slowdown or even reversal in the growth rate of the phage population following the dynamical trajectory derived in Ref. 13. Indeed, in our model we can represent this trajectory by the corresponding distribution of instantaneous growth rates Ω(*t*). Abrupt downturns (“bad times”) in our model are caused by external events, such as e.g. invasions of new predators or emergence of resistant strains, (see e.g. Ref. 24). In addition, the negative feedback between populations of the phage and its bacterial host^13^ can cause dramatic short term changes in the lytic growth rate. The last approximation behind the Eq. 1 is that “bad-times” are treated as singular events of undetermined duration, thereby ignoring the topic of the optimal rate for lysogen induction. We employ this simplification because the lysogenic state represents a long term commitment that can be broken only due to rare stochastic fluctuations or excessive DNA damage of the host^25^. This simplification is well justified for 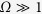 when the vast majority of phages were created during the previous time-step and thus the lysogenization frequency during this step directly determines the lysogenic fraction *x* of the entire population. Therefore, in this study we focus exclusively on the choice between lytic and lysogenic states during the infection, and not on the small spontaneous release of lytic phages from lysogens (of order 10^−5^ per generation per bacteria for phage λ^26^). A more extended and general formalism allowing for discussion of both entry and exit rates in two-state phenotypic model can be found in^18^,^27^,^28^,^29^.

Phages are known to combine stochastic and regulated strategies for entry to lysogeny in response to a variety of external and internal signals^30^. One example of such strategic response is provided by an increase in lysogenization frequency^31^ in response to reduced burst size when phages infect bacteria in a starved or stationary state^32^,^33^,^34^. Here the short term optimization criterion would predict full virulence as long as (1 − *p*) ⋅ Ω > 1, whereas the long term optimization (Eq. 2) would suggest a gradual increase of the optimal lysogeny frequency x* as Ω is reduced and/or *p* increases. In this study we do not consider the question of how phages can keep the lysogenic fraction of their population as close as possible to its Kelly-optimal value x*. Instead we concentrate on how x* itself depends on environmental and biophysical parameters.

### Regions of optimal temperate, virulent, or dormant phage strategies

To simplify our calculations, above we assumed that during bad times the entire lytic phage population dies off and that the lysogenic subpopulation does not change at all. Both assumptions can be relaxed by assuming a small but finite multiplicative ratio 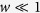 quantifying the collapse (but not complete extinction) of the lytic subpopulation during bad times. We also introduce the new parameter λ for the growth (or decline) rate of the lysogenic subpopulation defined by the replication rate of their bacterial hosts. The mathematical approach developed in our study requires λ to stay constant during both good and bad times in contrast to dramatic changes in growth rates of the lytic subpopulation.

In this more general case of our two-state model the logarithmic growth rate of the entire phage population is given by 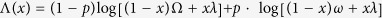 and the Kelly-optimal lysogenic fraction is given by (see Appendix for derivation).
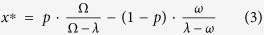


The important new result is the existence of a finite threshold for transition between purely lytic (virulent) and mixed lytic-lysogenic (temperate) strategies of phages. Assuming (quite realistically) that 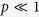 , 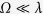 and 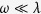 one gets the approximative relation x* = *p* − *ω*/λ, which predicts that a purely lytic strategy with x* = 0 is optimal when
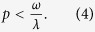
In other words, virulent phages thrive when the probability of environmental downturns (*p*) is smaller than the relative impact of such downturns on lytic and lysogenic subpopulations (*ω*/λ). On the opposite end of the spectra the growth advantage of the lytic over the lysogenic state under good conditions shrinks as Ω is decreased until it becomes comparable to λ. In this case phages (as smart investors) should allocate progressively larger portion of their population “capital” to the safety of the lysogenic state. When the time-averaged growth rate of a purely lytic population is less or equal than that of a purely lysogenic one, (1 − *p*)Ω + *pω* ≤ λ, the Kelly-optimal lysogenic fraction is equal to 1 instructing phages to permanently abandon the lytic strategy e.g. by transferring their genomes to plasmids. In between these two extremes, for moderate likelihoods of bad times *p*, and substantial good times lytic growth rates Ω, the temperate strategy will win. Thus the “well-temperate phage” from our title is the one whose lysogenization frequency within the duration of a lytic burst cycle is approximately equal to the likelihood of the lytic population collapse: x*≃; *p*.

**Figure 2:**
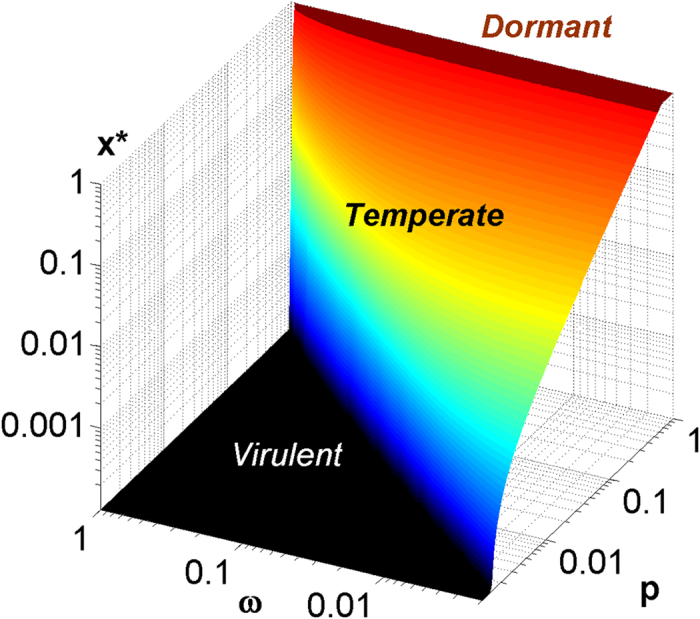
Kelly-optimal lysogenic ratio as a function of *p* - the probability of environmental downturn, and *ω* - the severity of population collapse during such downturn. Note the sharp boundary separating purely virulent (the blue region in the upper left corner) and temperate strategies. Equally sharp boundary separating temperate (0 < x* < 1) and dormant (x* = 1) strategies is less visible in this plot because of selection of colors. Here we used a two-state model with Ω = 3 and λ = 1 but other values of these parameters do not change the qualitative picture shown here.

The plot of the Kelly-optimal lysogenic fraction x* as a function of the probability - *p* and the severity - *ω* of population collapses during bad times is shown in Fig. 2.

One can further generalize our model from two-state environments to multiple or even continuous state environments. In this model the lytic population growth rate Ω(*t*) during a given time-interval *t* is drawn from an arbitrary probability distribution *π*(Ω(*t*)). The two-state model considered above corresponds to *π*(Ω(*t*)) = (1 − *p*)*δ*(Ω(*t*) − Ω) + *pδ*(Ω(*t*) − *ω*).

In the appendix we prove the existence of a unique Kelly-optimal strategy which is
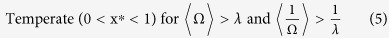

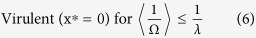

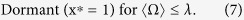
Here 〈〉 denotes the long-term time average calculated using *π*(Ω(*t*)). When 〈Ω〉 ≤ λ (Eq. (7)) there is no growth advantage of being lytic and hence the optimal choice is for the entire phage population to go dormant into the lysogenic state: x* = 1. On the other hand, when 〈1/Ω〉 ≤ 1/λ (Eq. (6)), the frequency and severity of lytic population collapses is not sufficient to justify even a marginal investment into “safe” lysogenic state. Hence the optimal choice in this limit is for the entire phage population to remain lytic: x* = 0. In between these two extreme scenarios the temperate strategy is optimal (Eq. (5)), and, as shown in the Appendix, the “well-temperate” (Kelly-optimal) lysogenic ratio x* is determined by numerical solution to
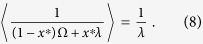


If the lysogenic subpopulation was completely stable, one would have the following paradox: the growth in the Kelly-optimal solution can only be larger or equal than the lysogenic growth rate λ. Thus, for λ = 1, the optimal temperate strategy would necessarily be non-stationary. This paradox does not exist if λ < 1. Such smaller growth of lysogenized hosts is in fact expected due to either cost of integrated prophages (see Ref. 13 for quantitative estimates) or predation of lysogens by other types of phages. In this case we expect the entire phage population to self-organize into a stationary state where over the longest times scale it neither shrinks nor grows: 〈log[(1 − x*)Ω + x*λ]〉 = 0.

### Independent, interconnected environments favor virulence

Besides previously discussed factors such as the frequency and the severity of environmental collapses, the choice between virulent and temperate strategies depends also on phages’ ability to access (e.g. by diffusion) multiple spatially-separated environments, which are fluctuating independently of each other.

In general, the access to multiple independently fluctuating environments favors the virulent strategy, in much the same was as the access to a well-diversified investment portfolio consisting of multiple independently fluctuating stocks reduces investor’s risk exposure^17^ and tempts to move his/her capital out of the safety of a low interest bank deposit. Analogous trends have been reported^21^ for seed germination of plants, also suggesting that the fast growing but risky strategy wins when offsprings can be spread between many independently fluctuating environments.

For phage populations, as their access to multiple independent environments increases, hedging of bets by the lysogeny becomes progressively less and less important. Eventually, in the limit of a large number of strongly interconnected environments one expects the purely lytic (virulent) strategy to win over any temperate strategy.

To quantify this common sense prediction in terms of phage biophysics and environmental parameters, we mathematically consider the case where the diffusion connects phage populations in *N* of independent yet statistically identical environments. We assume that at every time-step the diffusion distributes a fraction *γ* < 1 of the entire phage population equally across all environments.

Generally speaking, in the multi-environment model the diffusion constant *γ* takes the role of the collapse ratio *ω* in the single-environment model. Indeed, during severe environmental downturns when the entire lytic subpopulation is lost it is replenished by phages diffusing from other environments at the rate *γ*. Therefore, in our subsequent numerical simulations we set *ω* = 0.

The multiplicative growth ratio of the entire *lytic* subpopulation in all environments is given by 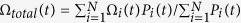. where *P*_*i*_(*t*) is the accumulated lytic subpopulation in the environment *i* and Ω_*i*_(*t*) is its growth ratio at time-step *t*. As before, it is equal to Ω with probability 1 − *p* and 0 with probability *p*. To estimate the overall growth, one needs to know the distribution of populations in all environments, which in turn is dependent on the spatiotemporal pattern of growths and collapses. Assuming the same lysogenic ratio *x* and lysogenic growth rate λ in all environments , the long-term logarithmic growth rate of the total phage population (both lytic and lysogenic) is given by the time average 〈log((1 − *x*)Ω_*total*_(*t*) + *x*λ)〉.

**Figure 3:**
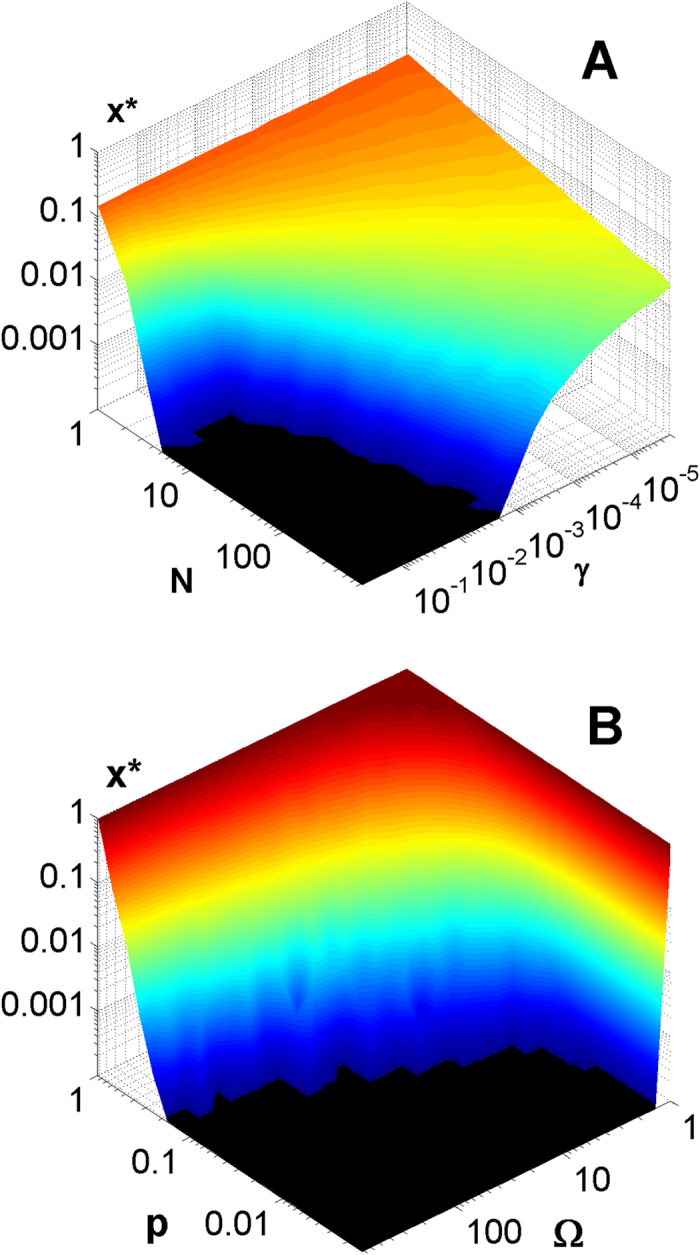
Kelly-optimal lysogenic ratio x* plotted as a function of the diffusion rate *γ* and the number of environments *N* for a two-state model with Ω = 3 and *p* = 0.1 (panel **A**) or as a function of the lytic growth ratio Ω and the probability of population collapse *p* for model with *γ* = 10^−4^ and *N* = 100. Note the sharp boundary to purely virulent strategy with x* = 0 (black area) which is optimal for large *γ* and *N*, as well as for large Ω and small *p*. The temperate strategy (colored area) is optimal in the opposite limit.

Numerical simulations allowed us to estimate the distribution of Ω(*t*) and to subsequently numerically solve the Eq. (8) (see “Model and Numerical Simulations” for more details) to self-consistently determine Kelly-optimal lysogenic fraction x*. Fig. 3A show how thus defined x* depends on the number of environments *N* and the diffusion constant *γ* in a model with Ω = 3 and *p* = 0.1. This figure quantifies the common-sense prediction that x* should decrease with both *N* and *γ*. The same overall trend was previously found in a similar model^21^ for plant seed germination and dispersal. In Fig. 3B we show that in the model with *γ* = 10^−4^ and *N* = 100 when the lytic growth ratio Ω goes up and/or the collapse frequency *p* goes down the optimal lysogeny frequency x* decreases and ultimately vanishes, indicating a transition to the purely virulent strategy. In contrast to a rather weak dependence of x* on Ω predicted for a single isolated environment (see Eq. (3)), our numerical results for multiple interconnected environments shown in Fig. 3B indicate that the fast lytic growth quantified by Ω strongly favors purely virulent strategy. Our simulations demonstrate the existence of a sharp boundary (see the border between colored and black areas in both panels of Fig. 3) above which the lysogeny is no longer required and pure virulence becomes the optimal phage strategy.

## Discussion

Sustainability of different strategies of phage predation has been theoretically explored before. Particular attention has been paid to the question of long-term sustainability of the pure virulent strategy^1^,^11^,^33^,^34^,^35^. At a first glance populations of lytic phages are inherently prone to collapses. Indeed, each infection leads to hundreds of new phage particles, which rapidly deplete the population of susceptible bacteria leading to the steady state with a low density of hosts prone to collapses^13^. Temperate phages, on the other hand, provide lysogens with the immunity against their own siblings and therefore are able to survive irrespective of host’s density. Models of interactions between temperate phages and their hosts have been considered by^34^,^36^,^37^. Stewart and Levin^13^ directly compared the resilience of temperate and virulent phage populations after their hosts were exposed to changes in nutrient levels. One of the important results of that study is that for substantial variations of nutrient availability temperate phages fare better than virulent ones. We have confirmed this earlier prediction using a very different type of model that takes into account not just nutrient variability but any other type of environmental downturns. Whenever temperate strategy beats virulent and dormant ones our approach allowed us to show that the optimal lysogeny frequency of a “well-temperate” phage resulting in the fastest population growth, has to be close to the probability for collapse of their local environments.

For pedagogical reasons, in our presentation above we assumed that the lysogenization frequency *x* has to stay the same throughout the duration of good environmental conditions. In other words, we ignored phages ability to actively sense the environmental state and to use this information to dynamically adjust the lysogenization frequency. However, our estimates can be directly extended to the case where phages can distinguish between multiple types of “good” environments each characterized by its growth rate and its own likelihood and intensity of collapse. If these environments occur randomly and independently from each other, the overall growth can be factorized and we predict phages would adjust the lysogenization frequency in each one of these environments to be given by Eq. (3) with parameters Ω, *p*, and *ω* characteristic of this particular environment. One example of this is provided by λ-phages collecting information about the nutritional state of the host and on whether it was simultaneously co-infected by other λ-phages^31^. This information is then processed by the phage to make lysis-vs-lysogeny decision that is in agreement with our predictions. That is to say, λ-phage’s lysogenization frequency is known to increase^31^ when they infect starved or multiple-infected hosts which both signal reduced prospects of lytic growth captured in our model by reduction in Ω and/or increase in *p*.

Optimal behavior of phages with respect to choice between virulent and temperate strategies depends on multiple extrinsic and intrinsic parameters such as phages’ burst size, host range, hosts’ availability, susceptibility, and average growth rate, the frequency and severity of environmental collapses, and finally phages’ ability to diffuse across multiple environments within their lifetime as an infectious particle. Our key predictions are: 1) The temperate phage strategy dominates when environmental downturns happen frequently, are severe, or when phages live in isolated and simple environments. 2) The virulent phage strategy gains the upper hand when the probability of downturns gets smaller, and/or collapses themselves are milder, and phages have access to multiple hosts or environments connected by diffusion (see Fig. 3B). This prediction is consistent with the empirical observation^10^ that virulent phages have systematically larger adsorption rates and shorter latency times than temperate phages.

Finally, our analysis suggests a simple explanation of why virulent mutants of temperate phages are not commonly found in the wild. Assuming that mutants are exposed to the same environmental risks as their ancestors we can calculate the reduction in the growth rate of a virulent mutant relative to its well-tempered ancestor:
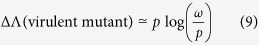
(see Methods for the exact formula and its derivation). This is a particular case of Bergstrom and Lachmann’s results^18^ relating fitness differences to Shannon entropy of the environment and the measure of organism’s information about the environment. In our case fitness loss of the virulent mutant compared to its optimally-tempered wild type ancestor is related to the information lost when the probability of environmental collapse - *p* is used as a proxy for its severity - *ω*.

The temperate strategy is optimal when *p* > *ω* (see Eq. (3)). Hence, a virulent mutant of a temperate phage has lower fitness than its ancestor:ΔΛ(virulent mutant) <0. Overcoming this fitness barrier by adjusting any of the other intrinsic properties of the phage such as e.g. its host range would require multiple simultaneous mutations and is, therefore, unlikely. This prediction of non-sustainability of virulent mutants of temperate phages is in agreement with the observation that protein families in known virulent phages have essentially no functional overlap with those in temperate phages^38^.

The bet-hedging approach introduced in our study focuses on random external shocks to the system at the expense of its intrinsic dynamics. Thus we treat the growth rates in a given environment as fixed and completely ignore the density-dependent feedback between populations of phages and their bacterial hosts^1^,^11^,^13^. Neglecting such feedback allowed us to obtain multiple mathematical insights that would be difficult to derive otherwise. However, this simplification may influence some of our predictions, in particular for “smart” phages capable of using the state of its host to predict availability of hosts in near future. Future work is needed to combine our bet-hedging formalism with density-dependent population dynamics.

## Methods

### Model and numerical simulations

Our model is updated in discrete time-steps. At each time-step the lytic subpopulation in each of the environments is either grows by a factor 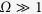 (with probability 1 − *p*), or collapses by a factor *ω* << 1 (with probability *p*). The lysogenic subpopulation is initially kept constant, and subsequently re-adjusted such that the selected fraction *x* of the total phage population in a given environment is assigned to the lysogenic state. In case of multiple independent environments (Fig. 3), the diffusion operates at each times-step by redistributing the fraction *γ* of the total phage population equally among all environments: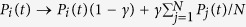. The simulations provide us with the numerical expression for the distribution of populations *P*_*i*_ across the environments and times. This distribution in its term determines the distribution *π*(Ω_*total*_(*t*)) of growth rates of the global phage population in all of the environments. The Kelly-optimal fraction x* is then found by numerically solving the Eq. (8) for the distribution *π*(Ω_*total*_(*t*)). After this we recalculate the distribution of *P*_*i*_ and *π*(Ω_*total*_(*t*)) for the new value of *x* = x*. This iterative process is repeated until the relative difference of x* during subsequent iterations is less than 1%.

### Kelly-optimal ratio for the two-state environmental model

In the general version of the two-state environmental model the long-term logarithm growth rate is given by Λ(*x*) = (1 − *p*) ⋅ log((1 − *x*)Ω + *x*λ) + *p* ⋅ log((1 − *x*)*ω* + *x*λ). Here Ω and *ω* are the growth rates of the lytic subpopulation under good and bad environmental conditions correspondingly, while λ is the constant growth rate of the lysogenic subpopulation. Taking the derivative with respect to *x* and setting it to 0 results in the following equation for x*
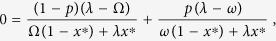
which can be further simplified to
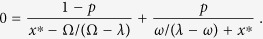
Grouping all the terms with 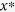 on one side results in the following expression for the Kelly-optimal lysogenic fraction:
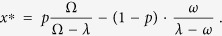


### Kelly-optimal ratio for the multi-state (continuous) environmental model

Here we consider a more general model in which the current growth rate Ω(*t*) of lytic subpopulation is not limited to just two values Ω and *ω* but is independently drawn from an arbitrary probability distribution *π*(Ω(*t*)). In this case the long-term logarithmic growth rate is given by
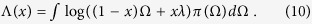
The Kelly-optimal lysogenic ratio 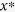 is determined by solving
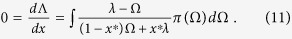
Note that the second derivative of 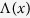 equal to
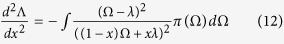
is always negative. The boundary conditions are given by *d*Λ/*dx*|_*x*=0_= 〈(λ − Ω)/Ω〉  = λ〈1/Ω〉 − 1 and *d*Λ/*dx*|_*x* = 1_ = 1 − 〈Ω〉/λ. Thus, as long as λ〈1/Ω〉 − 1 > 0 (or 〈1/Ω〉 > 1/λ ) and 1 − 〈Ω〉/λ < 0 (or 〈Ω〉 > λ) a unique solution for the Kelly-optimal lysogenic fraction x* between 0 and 1 exists.

When 〈Ω〉 ≤ λ there is no growth advantage (yet all the risks) of going lytic and hence the optimal state for the phage population is to be 100% lysogenic: x* = 1. On the other hand when 〈1/Ω〉 ≤ 1/λ the growth rate during bad times (small Ω) dominating this average is not low enough to justify even marginal “safety net investment” into the lysogenic state. Hence the optimal strategy for phage population in this case is to be 100% lytic: x* = 0.

A more concise way to write the equation for the Kelly-optimal lysogenic ratio can be derived by multiplying the Eq. (11) by 1 − x* and noticing that the numerator can be regrouped as (1 − x*)(λ − Ω) = λ − [(1 − x*)Ω + λx*]. Hence, the ratio under the integral of the Eq. (11) can be replaced with λ/[(1 − x*)Ω + x*λ] − 1 which gives
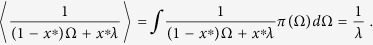
Hence the purpose of x* is to provide an “insurance” lower bound x*λ for the denominator when Ω is very small. This way the Kelly-hedged growth ratio Ω* = (1 − x*)Ω + x*λ satisfies 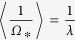. As derived above such insurance is necessary only when small values of Ω happen sufficiently frequently to make 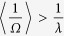.

For log-normally distributed Ω described by *π*(Ω) = exp( − (logΩ − *μ*)^2^/2*σ*^2^)/Ω/Norm the calculation of the parameter range for which temperate strategy is Kelly-optimal is especially simple. Indeed in this case *m*-th moment of Ω, 〈Ω^*m*^〉 = exp(*mμ* + *m*^2^*σ*^2^/2). Thus, in order to have 〈Ω〉 λ and 〈Ω^−1^〉 1/λ one needs log λ − *σ*^2^/2 < *μ* < log λ + *σ*^2^/2. Adding *σ*^2^/2 to all sides of this double inequality one gets a condition for optimality of the temperate strategy as
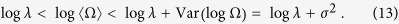
In other words, in order for the temperate strategy to beat the virulent and the dormant ones, the logarithm of the average growth rate in the lytic state has to be within one standard deviation of log Ω above the logarithm of the growth rate of the lysogenic state. This is possible either when these two growth rates are very close to each other or when the variability of log Ω is very large. Note that this equation can be realized when λ < 1, 〈Ω〉 1, and the overall phage population is stationary: (1 − x*)〈Ω〉 + x*λ = 1.

### Fitness advantage of the Kelly-optimal strategy over purely virulent strategy in the two-state environmental model

To quantify the fitness advantage of the Kelly-optimal strategy over purely virulent strategy in the most general formulation of the two-state environmental model let’s recall that
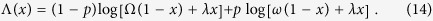
Thus the fitness advantage 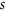 of the Kelly-optimal over purely virulent strategy can be written as
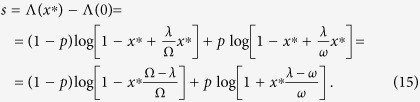


Recalling, that according to Eq. (10)
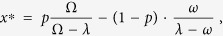
one can further simplify the expression for 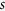 to be exactly equal to
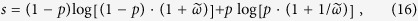
where 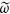 is a shorthand for
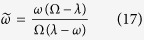
Perhaps the most concise expression for *s* is in terms of the Kullback-Leibler relative entropy *S*_*K* − *L*_ between bimodal probability distributions *p* and *q*:
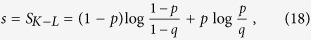
where the “probability” 0 < *q* < 1 is given by the ratio
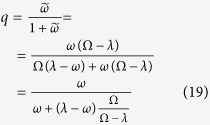
In the limit 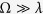 one can further approximate
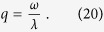


## Additional Information

**How to cite this article**: Maslov, S. and Sneppen, K. Well-temperate phage: optimal bet-hedging against local environmental collapses. *Sci. Rep.* **5**, 10523; doi: 10.1038/srep10523 (2015).
